# Detection of Tumor Cell-Specific mRNA and Protein in Exosome-Like Microvesicles from Blood and Saliva

**DOI:** 10.1371/journal.pone.0110641

**Published:** 2014-11-14

**Authors:** Jieping Yang, Fang Wei, Christopher Schafer, David T. W. Wong

**Affiliations:** School of Dentistry, University of California Los Angeles, Los Angeles, California, United States of America; Queen Mary University of London, United Kingdom

## Abstract

The discovery of disease-specific biomarkers in oral fluids has revealed a new dimension in molecular diagnostics. Recent studies have reported the mechanistic involvement of tumor cells derived mediators, such as exosomes, in the development of saliva-based mRNA biomarkers. To further our understanding of the origins of disease-induced salivary biomarkers, we here evaluated the hypothesis that tumor-shed secretory lipidic vesicles called exosome-like microvesicles (ELMs) that serve as protective carriers of tissue-specific information, mRNAs, and proteins, throughout the vasculature and bodily fluids. RNA content was analyzed in cell free-saliva and ELM-enriched fractions of saliva. Our data confirmed that the majority of extracellular RNAs (exRNAs) in saliva were encapsulated within ELMs. Nude mice implanted with human lung cancer H460 cells expressing hCD63-GFP were used to follow the circulation of tumor cell specific protein and mRNA in the form of ELMs *in vivo*. We were able to identify human GAPDH mRNA in ELMs of blood and saliva of tumor bearing mice using nested RT-qPCR. ELMs positive for hCD63-GFP were detected in the saliva and blood of tumor bearing mice as well as using electric field-induced release and measurement (EFIRM). Altogether, our results demonstrate that ELMs carry tumor cell–specific mRNA and protein from blood to saliva in a xenografted mouse model of human lung cancer. These results therefore strengthen the link between distal tumor progression and the biomarker discovery of saliva through the ELMs.

## Introduction

Human saliva is a clear, slightly acidic (pH 6.0–7.0) biofluid known to contain a diverse range of proteins [1], over 3000 unique mRNAs [1], [2], [3], and 700 bacterial species [4]. In recent years, a number of investigations evaluating the constituency of oral fluid have discovered that it may actually reflect an individual's physiological condition. In fact, multiple groups have identified saliva-based proteomic, transcriptomic, and microbiological markers for Sjögren's syndrome, inflammatory bowel disease, and even cancers [5], [6], [7], [8], [9], [10], [11], [12], [13]. Although promising, the novelty of using saliva as an effective evaluator of local and systemic health has not found widespread acceptance. Significant skepticism remains regarding how these unique molecular indicators are developed in saliva.

Exosomes are small, lipid-bound, spherical structures measuring approximately 30 to 100 nm in diameter [14], [15]. Randomly formed from the invagination of intracellular vesicles, exosomes often contain biologically active host cell lipids, proteins, miRNAs, mRNAs, ncRNAs, and other cellular constituents [14], [16], [17], [18], [19], [20], [21]. Microvesicles containing similar host cell biomolecules and heterogeneous in size may also be formed by the budding-off the cellular membrane [22], [23]. Majority of vesicles isolated from body fluids are referred as exosomes based on their exosomal protein markers [24], [25]. However, the microvesicles do be co-isolated using current available purification method [24], [25]. We, therefore, collectively refer these vesicles as exosome-like microvesicles (ELMs) here. ELMs are known to shed continuously from multiple cell types including: hematopoietic, intestinal epithelial, Schwann, fat, neuronal, fibroblasts, and several tumor cell lines [26]. Many types of cancer cells release ELMs and tumor-derived ELMs carry a wide range of nucleic acids, including miRNA, mRNA, ncRNA and DNA [21]. ELMs containing these nucleic acids have been shown to reflect the genetic status of tumor, and be able to travel to distant site and transfer their cargo to the recipient cells and to induce phenotypic changes [27], [28], [29]. Previous investigations have revealed that tumors are often the primary source of circulating membrane vesicles and increased amount of tumor derived protein, RNA and DNA were found in the blood of cancer patients [30], [31], [32], [33].

In addition to their presence in blood, ELMs are also present in urine, saliva, breast milk, malignant and pleural effusions, synovial fluid, epididymal fluid, and amniotic fluid [21], [26]. Therefore, tissue-specific exosomes with their constituent tissue-specific biomarkers can serve as a biomarker source for the diagnosis, prognosis, and monitoring of disease [34], [35], [36].

Recent evidence has emerged describing a role for ELMs in the processes that govern the induction of discriminatory salivary biomarkers [17], [37], [38]. However, the mechanisms underpinning the etiology and biogenesis of saliva-based biomarkers have not been clearly explained. Understanding how these markers come to exist in oral fluids will both shed light on the body's capacity for extracellular communication and help credential salivary biomarkers as an acceptable mode for personalized medical assessment.

In this study, we demonstrated that exRNA in cell-free saliva were largely encapsulated and protected from degradation by ELMs. A novel human lung cancer mouse xenograft model in which implanted human lung cancer H460 cells consistently express exosomal marker hCD63-GFP was developed to study whether ELMs contribute to the emergency of discriminatory salivary biomarkers during systemic disease progression. We were able to identify hCD63-GFP positive ELMs and human GAPDH mRNA in hCD63 positive ELMs in the blood and saliva of tumor bearing mice. Our evidence indicates that ELMs play a critical role in this process by acting to protect and transport tumor-specific molecular information throughout the vasculature, as well as in bodily fluids. This study represents a substantial discovery, describing the induction of discriminatory saliva-based biomarkers, Understanding these phenomena may facilitate the development of novel strategies for diagnostics, monitoring, and therapeutics.

## Materials and Methods

### Ethics Statement

Human saliva samples were obtained from healthy volunteers under the institutional review board protocol (IRB#10-000431) approved by the University of California Los Angeles IRB. Written informed consent forms were obtained from all participants.

All mouse procedures were approved by the UCLA Animal Research Committee in compliance with the Association for Assessment and Accreditation of Laboratory Care (AAA-LAC) International.

### Saliva Collection and Salivary ELMs Isolation

Unstimulated saliva samples were obtained and processed according to previously established protocols [2]. ELMs were isolated using Exoquick precipitation solution (System Biosciences, Inc.). Three hundred microliters of cell-free saliva was thoroughly mixed with an equal volume Exoquick solution, incubated at 4°C overnight, and centrifuged at 1500×*g* for 15 min at 4°C. The pellets were then resuspended in water [39] and used for RNA isolation and immunoblotting via electron microscopy (EM).

### Isolation of RNA from Saliva and ELMs

Cell-free saliva, salivary ELMs, and ELM-depleted saliva supernatant were treated with RNase cocktail (final concentration 100 U/ml) with or without 1% Triton X-100 (Tx) at room temperature for 20 min. RNA was extracted from these processed samples using the RNeasy Mini Kit (Qiagen) according to the manufacturer's instructions. The isolated RNA was quantified using the RiboGreen RNA quantification Kit (Invitrogen) and analyzed by reverse transcription PCR (RT-PCR) followed by quantitative PCR (qPCR).

### Cell Culture

The human non-small cell lung cancer cell line H460 and mouse Lewis lung cancer cell line LLC1 were obtained from ATCC. Cells were grown in DMEM medium with glutamax-I supplemented with 10% (v/v) FBS and Pen/Strep in an atmosphere of 95% air and 5% CO_2_ at 37°C. H460 cells were transfected with the plasmid encoding hCD63-GFP, pCT-CD63-GFP (SBI, USA). Stable H460-hCD63-GFP cells were selected and maintained in puromycin-containing medium at 1 µg/ml. Dimethyl amiloride (DMA) (Sigma, MO) was used to inhibit ELM secretion in H460-hCD63-GFP cells [40]. Cells were treated with 1 µmol or 10 µmol DMA for 48 h, and the amount of ELMs secreted into medium in the presence of DMA was determined and compared to untreated controls using an acetylcholinesterase activity assay. The trypan blue exclusion assay was used to determine the cytotoxity of DMA using a Beckman Coulter Vi-Cell (Beckman Coulter Inc., CA).

### Western blotting

H460 hCD63-GFP and H460 cells were grown in serum-free DMEM supplemented with 1% non-essential amino acids for 48 h. ELMs were collected from the conditioned media and lysed along with both H460 hCD63-GFP and H460 cells. The H460 hCD63-GFP and H460 cell and ELM lysates (5 µg protein) were denatured at 95°C for 10 min and loaded onto 12% sodium dodecyl sulfate–polyacrylamide gel electrophoresis (SDS-PAGE). Proteins were transferred to a nitrocellulose membrane and probed with rabbit anti-GFP HRP-conjugated antibody (Invitrogen). ELMs were isolated from human saliva using exoquick precipitation (System Biosciences, Inc.). Exoquick precipitated pellets were lysed and then separated by SDS-PAGE. Proteins were transferred to a nitrocellulose membrane and probed with rabbit anti-CD63 (Santa Cruz Biotechnology, Santa Cruz, CA) followed by an incubation with a secondary antibody (Invitrogen).

### Isolation and Quantification of ELMs from Condition Medium

ELMs were prepared using differential ultracentrifugation methods, as described previously [41]. Briefly, cell-free conditioned media was centrifuged at 10,000×*g* for 30 min at 4°C, then at 100,000×*g* for 2 h at 4°C. The ELM pellet was washed and resuspended in PBS. The Amplex Red acetylcholinesterase assay kit was used to measure the total amount of ELMs according to the manufacturer's protocol (Invitrogen, USA).

### Characterization of ELMs Using Electron Microscopy

Isolated ELMs were loaded onto carbon-coated grids; fixed in 2% paraformaldehyde; washed in PBS; and immunolabeled with anti-hCD63 (BD Biosciences, US) or anti-CopGFP primary antibodies (Evrogen, Moscow, Russia) and goat anti-mouse or rabbit IgG coupled to 10-nm or 15-nm gold particles (Sigma, US). The grids were post-fixed in 2.5% glutaraldehyde, washed, and contrasted with 2% uranyl acetate, as previously described [42]. The ELMs were then examined with a JEOL 100GX transmission electron microscope (JEOL USA, Inc. Peabody, MA) (Electron Microscopy Service Center, Brain Research Institute, UCLA).

### Mouse Lung Cancer Xenograft Model

Male athymic BALB/c nude mice were obtained from Charles River (MA, USA) and weighed 20–22 g at the start of the experiments. The mice were housed in sterilized filter-topped cages and maintained in sterile conditions. A total of 1×10^6^ H460 hCD63-GFP cells, or 100 µl saline were injected into the left chest cavity of nude mice. Briefly, mice were anesthetized using 1–3% isoflurane in oxygen from a precision vaporizer. Mice were placed in the right lateral decubitus position. One hundred microliters of cells or saline were injected slowly into the left intercostal space at the dorsal mid-axillary line just below the inferior border of the scapular using a 30-gauge needle attached to a 1-ml syringe. The needle was advanced approximately 5 mm through the chest wall into the pleural space. After injection, the needle was retracted and mice were turned to the left lateral decubitus position for recovery. Some tumor-bearing mice were treated with daily intrapleural injection of 1 µmol/kg DMA (Sigma) or PBS control for 1 week before sacrifice [43].

### Collection of Mouse Saliva, Blood, and Tumor Tissue

Twenty days after tumor implantation, saliva was collected and mice were sacrificed. Mild anesthesia was induced by intramuscular (IM) injection of 1 µl/kg body weight of a solution containing 60 mg/ml ketamine (Phoenix Scientific, St. Joseph, MO) and 8 mg/ml xylazine (Phoenix Scientific, CA). To stimulate saliva secretion, mice were subcutaneously injected between the ears with pilocarpine (0.05 mg pilocarpine/100 g body weight). Collection was completed in 20 min using a micropipette, and samples were immediately placed in pre-chilled 1.5-ml microcentrifuge tubes. Samples were centrifuged at 3500 rpm for 30 min at 4°C and supernatant was collected and stored at −80°C until analysis. Blood was collected in BD vacutainer tubes containing clot activator (BD Biosciences, CA) and centrifuged at 1000×*g* for 10 min after mice were sacrificed. Salivary glands and tumor tissues were removed from mice, snap-frozen in liquid nitrogen, and stored at −80°C.

### Detection of hCD63/GFP-Positive ELMs Using EFIRM Technology

The human hCD63/GFP-positive ELMs were quantified using immuno-adsorption and an electrochemical sensor detection technology, called electric field-induced release and measurement (EFIRM) [44], [45]. Briefly, saliva, serum or conditioned media were centrifuged at 1000 rpm at 4°C for 10 min. The cell-free supernatant was further centrifuged at 3500 rpm at 4°C for 30 min, to eliminate cellular debris. Five microliters of streptavidin-coated magnetic beads (Invitrogen, USA) were mixed with 1 µl of biotinylated anti-human CD63 (hCD63) antibody (Ancell, USA) on a HulaMixer Sample Mixer (Invitrogen) for 30 min at room temperature. Then, 1 ml of conditioned medium, or 10 µl of saliva or serum diluted in 990 µl of casein-PBS (Invitrogen, USA), was incubated with the anti-hCD63-conjugated magnetic beads for 2 h at room temperature to form ELMs–magnetic bead complexes. Beads were then washed twice with casein-PBS and incubated with rabbit anti-GFP HRP-conjugated polyclonal antibody for 1 h at room temperature. After incubation, the beads were washed twice with casein-PBS and accumulated onto an electrochemical sensor by applying a magnetic field [44], [45].

### Confocal Microscopy

The tumor sections were examined using a Leica TCS-SP2 confocal microscope with Plan APO 63×1.4 NA oil objective lens and LCS confocal software.

### RT-qPCR Assay

RT-PCR followed by separate qPCR (hereafter termed RT-qPCR) was performed to detect salivary mRNAs. Multiplex RT-PCR preamplification of three mRNAs was performed using a SuperScript III platinum qRT-PCR System (Invitrogen) with a pool of outer primer sets, and conducted using a GeneAmp PCR-System 9700 (Applied Biosystems) with a fixed thermal-cycling program. SYBR Green qPCR was performed to quantitatively detect the expression levels of salivary mRNAs. The qPCR sample was prepared by combining 2× qPCR Mastermix (Applied Biological Materials), inner primers (900 nmol/l; Table S1 in Supporting information S1), and 2 µl cDNA template. The total volume of each reaction was 10 µl, adjusted with nuclease-free water. The qPCR associated with melting-curve analysis was conducted using an AB-7500HT System (Applied Biosystems) with a fixed thermal-cycling program. Each gene was tested in triplicate for all samples, including the negative control, in which the cDNA template was the product of the RT-PCR preamplification negative control.

### Human GAPDH Nested RT-PCR Detection

hCD63-positive ELMs from mouse saliva or blood were adsorbed onto anti-human anti-CD63 antibody-coated magnetic beads, as previously described [44], [45]. Beads were washed twice with PBS and resuspended in 10 µl TE buffer (Ambion, USA). Human GAPDH RNA was reverse transcribed using 4 µl of ELM-bead complex and the SuperScript III One-Step RT-PCR System (Invitrogen, USA). Nested PCR reactions for human GAPDH were performed using the following primer sets:

5′-TCAAGTGGGGCGATGCTGGC-3′ and 5′-TGGGGGCATCAGCAGAGGGG-3′, 5′-GCTGGCGCTGAGTACGTCGT-3′ and 5′-CCTGCAAATGAGCCCCAGCC-3′. PCR clean-up was performed using ExoSAP-IT after the initial RT-PCR reaction (Affymetrix, CA, USA). Native -PAGE (15%) was used to reveal the 72-bp human GAPDH PCR product.

### Statistical Analysis

All statistical analyses were conducted using the IBM SPSS Statistics version 21; mean values, and standard deviations were calculated using descriptive statistics. One-way analysis of variance, two-sample *t* tests were used for testing the difference between expression values. *P* values <0.05 were considered significant. Post-hoc comparisons were conducted by using Tukey's analysis. Data are expressed as mean ± SD.

## Results

### Salivary ExRNAs are Predominantly Contained Within ELMs

**Figure 1:**
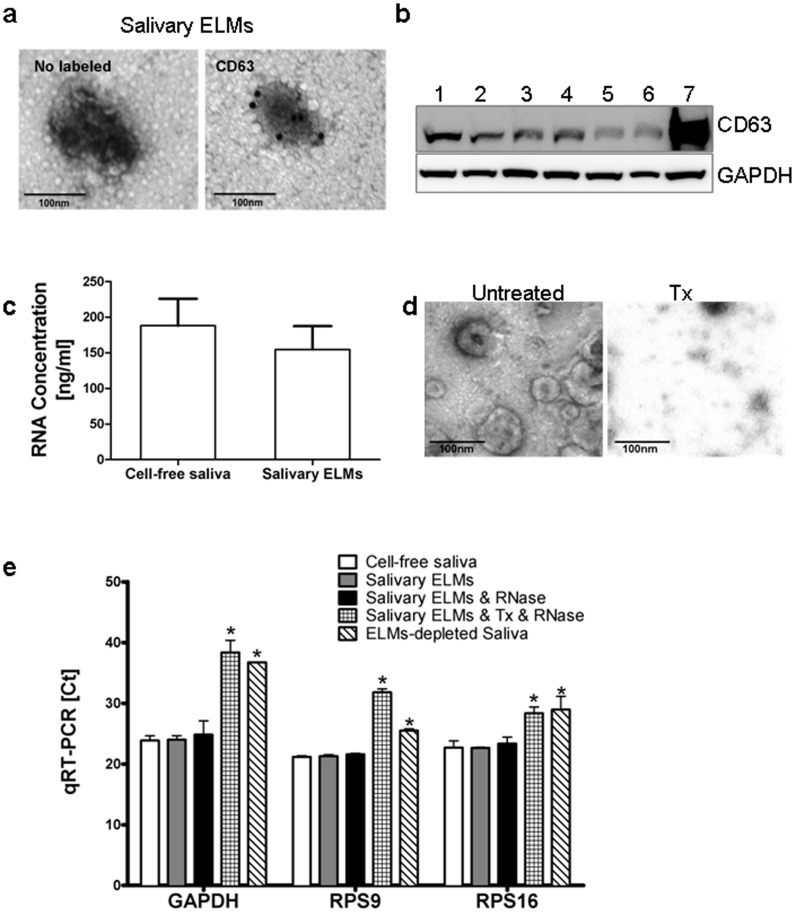
Salivary RNAs reside within salivary ELMs. (a) Representative electron microscopy images of salivary ELMs, and anti-CD63 immunogold-labeled ELMs in human saliva. (b) Western blot analysis of salivary ELMs using antibodies against human CD63 and GAPDH. Lane 1 to 7 are protein extracts of the salivary ELM pellets obtained from seven donors using Exoquick precipitation. (c) Ribogreen RNA quantitation from seven donors. Error bars represent means ± SD. (d) Representiave electron microscopy images of salivary ELMs and salivary ELMs treated with Tx. (e) RT-qPCR results of three reference genes from the following samples: cell-free saliva; salivary ELMs; salivary ELMs treated with RNase; salivary ELMs treated with Tx and RNase and ELM-depleted saliva. Data are expressed as means ± SD. *P<0.05, statistically significant difference from the saliva group.

Previous studies have demonstrated that cell-free saliva supernatant contains more than 3000 different mRNA species [1], [2], [3], and the exRNAs remain stable unless they are exposed to detergents [46]. Lipidic vesicles, exosomes, protected the breakdown of exRNAs in saliva [42]. To evaluate whether all exRNAs in saliva are subject to this type of vesicular protection, we compared exRNAs in cell-free saliva and salivary ELMs. We first isolated salivary ELMs from the cell-free saliva of seven healthy subjects, which were characterized by the expression of exosomal surface marker CD63 (Fig. 1a, b). exRNAs in salivary ELMs and cell-free salivary were quantified and found to range from from 74 to 300 ng/ml and 87 to 350 ng/ml, respectively, with no statistically significant difference (Fig. 1c). Consistent with previous observation [42], the exRNAs in salivary ELMs is protected from RNase degradation. Quantitative PCR analysis of extracted RNAs revealed no notable differences in *C*_t_ values of the reference genes GAPDH, ß-actin, or RPS9 between cell-free saliva, salivary ELMs, and salivary ELMs with RNase treatment (Fig. 1e). 1% Triton X-100 (Tx) solution compromised structural integrity of salivary ELMs (Fig. 1d). ELMs with Triton X-100 plus RNase treatment or ELMs-depleted saliva exhibited substantially higher *C*_t_ values of reference genes GAPDH, ß-actin, or RPS9 (Fig. 1e) These results indicate that exRNAs in cell-free saliva are largely encased within salivary ELMs.

Next, we sought to compare the transcriptome content of salivary ELMs vs. cell-free saliva using microarray expression analysis (Affymetrix HU133 plus 2.0). Approximately 2938 and 2040 mRNA transcripts were detected in cell-free saliva and isolated salivary ELMs respectively. Categorizing each group ontologically revealed similar mRNA profiles (Fig. S1a in Supporting information S1). Eight mRNAs were abundant across each data set (Fig. S1b in Supporting information S1). These overlapping molecular characteristics suggest that the transcriptomic constituency of saliva may exclusively reside within ELMs, a structure whose integrity is critical for the stability of salivary RNA.

### Establishment of an ELM-Labeling Xenograft Lung Cancer Mouse Model

**Figure 2:**
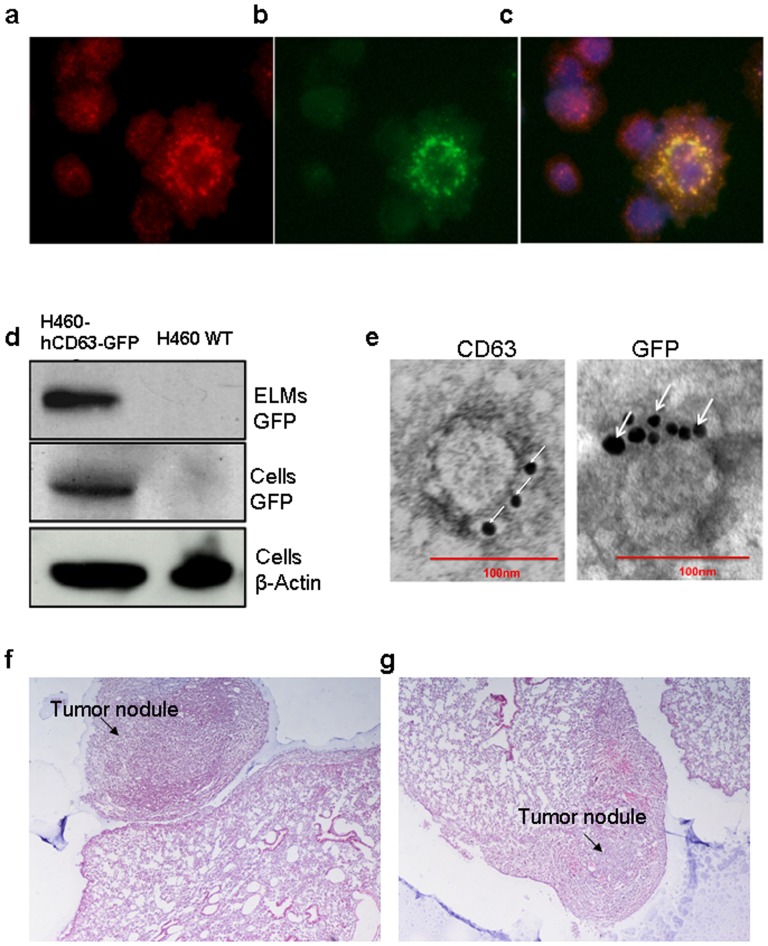
Establishment of a human lung cancer xenograft mouse model. To generate our xenograft lung cancer mouse model, H460 human lung cancer cells that stably express hCD63-GFP were orthotopically injected into male athymic BALB/c nude mice. CD63 immunolabeled (a, red) and GFP-postive (b, green) H460 CD63-GFP cells are shown. (c) Merge image of CD63, GFP, and DAPI staining. (d) Anti-CD63 and anti-GFP Western blot of ELMs and cell lysates from H460 CD63-GFP and H460 cells. (e) Electron microscopy images of anti-CD63-labeled and anti-GFP-labeled ELMs isolated from the conditioned medium of H460 CD63-GFP cells. Scale bar  = 100 nm. (f, g) Nude mice were intrapleurally injected with 1×10^6^ H460 hCD63-GFP cells. H&E staining of pulmonary tumor tissue shows (f) tumor nodule attached to the pleural surface and (g) tumor nodule in the lung parenchyma.

To help determine whether ELMs function as a conduit for long-range transportation of tissue-specific biomolecules, we labeled and tracked tumor-derived ELMs *in vivo*. We implanted nude mice with H460 human non-small cell lung cancer (hNSCLC) cells that stably expressed a well-known exosomal surface protein, hCD63 [20] fused to GFP (Fig. 2a–d). Prior to implantation, GFP-labeled H460-derived ELMs were confirmed by Western blotting and immuno-electron microscopy (Fig. 2d, e). The presence of tumors 20 days after implantation were confirmed by H&E section showing tumor nodule attached to the pleural surface and tumor nodule in the lung parenchyma (Fig. 2f, g). Together, these results confirm the establishment of human lung cancer xenograft mouse model with trackable tumor cell derived ELMs.

### Tumor-Specific mRNA in Blood and Salivary ELMs from Tumor-Bearing Mice

**Figure 3:**
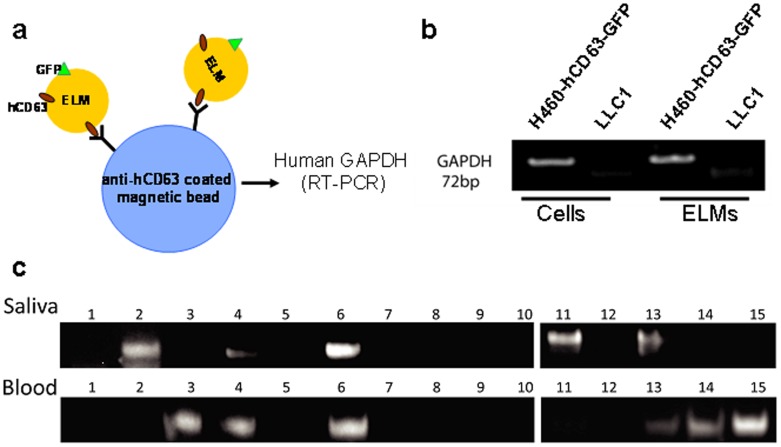
Detection of human GAPDH mRNA in tumor-bearing mice. (a) Illustration of anti-hCD63-coated magnetic beads used to isolate hCD63-positive ELMs from saliva, and blood. (b) This native PAGE analysis depicts the 72-bp nested RT-PCR products for human GAPDH from H460-hCD63-GFP cells, LLC1 cells, and (c) the blood and saliva of 15 H460-hCD63-GFP tumor-bearing or control mice.

With our *in vivo* model in place, we chose to explore its ability to determine the capacity of ELMs to shuttle tumor-specific mRNAs throughout the body. To effectively employ this model, we found it imperative to identify ELM-derived transcriptomic indicators of our implanted tumor cells. Given that GAPDH mRNA is abundant in ELMs [47], we elected to pursue it as a potential marker of ELMs exRNA transport. Using anti-hCD63 magnetic beads, we captured hCD63-positive ELMs from both the blood and the saliva of mice implanted with human H460-hCD63-GFP cells (Fig. 3a). To assess the specificity of our primers, we compared human GAPDH RT-PCR results on RNA extracted from H460-hCD63-GFP cells, murine Lewis lung carcinoma LL2/LLC1 cells, and their respective ELMs. Nested RT-PCR revealed that only H460 hCD63-GFP cells and their ELM derivatives were positive for human GAPDH mRNA (Fig. 3b). These results suggest that human GAPDH mRNA is an acceptable marker to detect H460 hCD63-GFP tumor cell-specific mRNA carried by hCD63-positive ELMs.

Next, we evaluated the efficacy of human GAPDH mRNA as an acceptable indicator of ELM-mediated exRNA transportation in our mouse model. Previous works report that mice that have been intrapleurally implanted with 1×10^6^ H460 cells subsequently developed lung tumor nodules within 20 days post-injection [48]. Accordingly, human GAPDH was detected in the blood and salivary ELMs of experimental mice within 20 days of implantation (Fig. 3c and g). 40% and 33% of all tumor-bearing mice carried the human GAPDH mRNA in their blood and salivary ELMs respectively (Fig. 3c; Table 1). These findings suggest that ELMs present in blood and saliva hold tissue-specific, viable, transcriptomic information, and support the hypothesis that ELMs might serve as potential targets of biomarker discovery and development.

**Table 1:** Detection of human GAPDH mRNA in saliva and blood from mice bearing hCD63-GFP tumors.

Mouse ID	Blood	Saliva
**1**	**ND**	**ND**
**2**	**ND**	**D**
**3**	**D**	**ND**
**4**	**D**	**D**
**5**	**ND**	**ND**
**6**	**D**	**D**
**7**	**ND**	**ND**
**8**	**ND**	**ND**
**9**	**ND**	**ND**
**10**	**ND**	**ND**
**11**	**ND**	**D**
**12**	**ND**	**ND**
**13**	**D**	**D**
**14**	**D**	**ND**
**15**	**D**	**ND**

### Detection of Tumor-Specific Pprotein in the Blood and Salivary ELMs of Tumor-Bearing Mice

**Figure 4:**
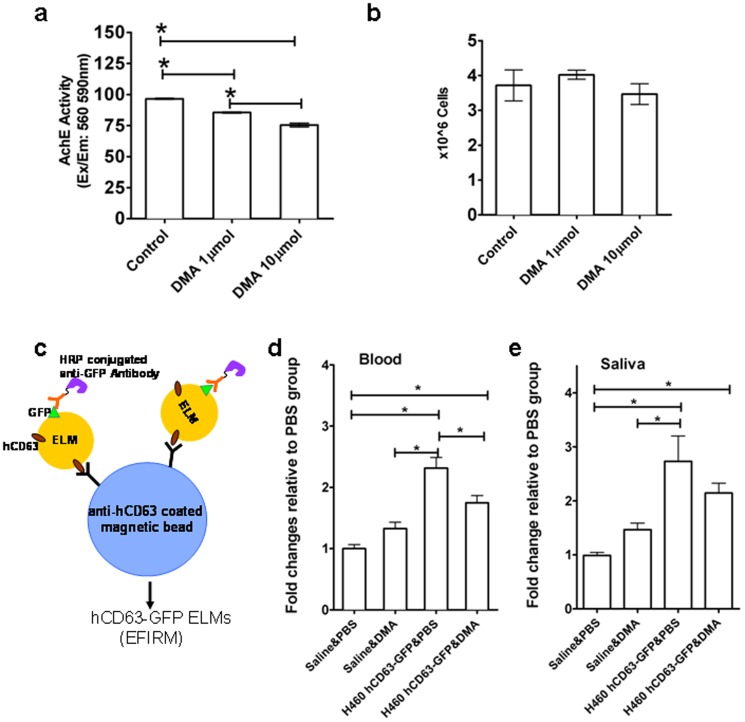
hCD63-GFP-positive ELMs in saliva and blood from tumor-bearing mice. (a) Acetylcholinesterase quantification of ELMs from the conditioned media of H460-hCD63-GFP cells treated with 1 µmol or 10 µmol DMA for 48 h. (b) Trypan blue exclusion assay of H460-hCD63-GFP cells after 48 h DMA treatment. (c) Illustration of the measurement of hCD63-positive ELMs in saliva and blood using EFIRM technology, allowing the detection of hCD63-GFP positive ELMs by electrochemical sensors. Samples of (d) blood and (e) saliva from control or tumor-bearing mice treated with PBS or DMA for 1 week and assayed for hCD63-GFP-positive ELMs using EFIRM technology. *N* = 9–11 per group; *P<0.05, statistically significant differences.

After demonstrating that ELMs contain tissue-specific exRNA molecules, we further probed these microvesicles to determine the likelihood that they contain additional molecular constituents. We focused on the proteomic content of hCD63-GFP-positive ELMs from saliva and blood. In doing so, we employed EFIRM technology (Fig. 4c) [45] to evaluate the blood and salivary hCD63-GFP-positive ELMs from H460 hCD63-GFP tumor-bearing mice exposed to either the ELMs secretion inhibitor dimethyl amiloride (DMA) [43] or PBS. Compared with PBS, DMA-treated H460 hCD63-GFP cells exhibited a non-cytotoxic, dose-dependent decrease in the ELM concentration of conditioned media (Fig. 4a, b). These results were echoed *in vivo* when, after 7 days of DMA or PBS treatment, the concentration of blood hCD63-GFP-positive ELMs was significantly decreased in DMA-treated mice compared with control animals (Fig. 4d). Salivary hCD63-GFP-positive ELMs was lower in DMA treated mice but not significant (Fig. 4e).

Collectively, these outcomes suggest a role for ELMs as conduits of tissue-specific molecular information. Furthermore, this finding highlights the concerted utility of EFIRM as a diagnostic tool and saliva as a diagnostic medium.

## Discussion

Discovering saliva-based biomarkers of oral and systemic disease [1], [2] has suggested the possibility of a paradigm shift in the field of molecular diagnostics. Identifying and validating disease-specific molecules in oral fluids could be of great interest to scientists and clinicians alike, and may facilitate the development of early disease diagnostic procedures and screening programs worldwide. Despite the inherent significance here, the mechanism by which markers of distal pathologies come to exist in salivary secretions currently eludes us. Here we show that small secretory lipidic vesicles called ELMs may have a substantial role in contributing to this phenomenon. We demonstrate that ELMs, secreted from distal tissues, encase and protect cell-specific RNAs and proteins within extracellular environments, including blood and saliva. Overall, our results suggest that ELMs play a significant part in transmitting biochemical information from remote cell populations to the oral cavity.

In agreement with our evidence, a recent publication showed that seven validated pancreatic cancer-specific mRNA markers and GAPDH have been detected in mouse salivary and blood-derived exosomes in a pancreatic cancer mouse model, and some of these mRNA markers were abolished when tumor cell exosome biogenesis was blocked [38]. Another interesting study found that mice bearing melanoma tumors have overlapping transcriptomic signatures in two tissues: the tumor itself, and the salivary gland [49]. Yet, it was concluded that salivary transcriptomic regulation was achieved through tumor cell-released mediators, such as growth factors and other inducers [49].

However, whether the induction of salivary molecular signatures were disease specific biomolecules directly shuttled into saliva through ELMs, or the result of a synergistic interplay between salivary glands and tumor-derived mediators, including growth factor and ELMs, remain largely unknown. Our mouse model was injected with human H460-hCD63-GFP lung cancer cells, which subsequently produced species-specific CD63 proteins and GAPDH mRNA, the very markers we detected and reported in blood and saliva. Accordingly, identification of these unique markers in distant tissues and fluids most likely happens after their excretion from xenograft hosts. Moreover, the appearance of saliva-based protein marker in our model showed a small decrease upon inhibition of ELM secretion, detracting from theories suggesting other means of regulation (Fig. 4e).

We however could not completely exclude the possibility that some of them were from metastatic tumor cells. To the best of our knowledge, no evidence of oral metastasis was seen in this widely used NCI-H460 human tumor xenograft model by previous publication [31], [48]. In addition, we did not observe lung metastasis at oral cavity by gross examination in this study. Therefore, we are inclined to conclude that H460-hCD63-GFP tumor-derived ELMs played a formative role in the generation of salivary biomarkers in our model.

Our present results in combination with previous reports demonstrate that ELMs thwart the enzymatic degradation of RNAs by providing a protective milieu [42], [50], [51], [52]. However, previous publication noted an ancillary mechanism of extracellular RNA transport by insinuating that circulating RNAs are associated with and sheltered from destruction by large protein complexes called ribonucleoproteins [53]. We have previously shown that approximately 30% of most common salivary exRNAs contain AU-rich elements (ARE) in their 3′ untranslated regions, which complex with ARE binding protein and protect salivary exRNAs from degradation [54]. In this study, 69% exRNAs identified from saliva of healthy individuals were present in salivary ELMs (Fig.S1a in Supporting information S1). We therefore speculate that most salivary exRNAs reside within ELMs. However, we cannot rule out the possibility of ribonucleoprotein contamination in our ELM samples. Our method of isolation employed the commercially available Exoquick kit to precipitate ELMs from bodily fluids [39], a technology that is relatively new and has not been thoroughly characterized [39]. Therefore, we cannot conclude that our ELM preparations were ribonucleoprotein-free, which may have affected our results. To overcome this issue, future experiments should employ ultracentrifugation-based ELM isolation protocols. Although this procedure requires larger volumes of fluid [42], it is the gold standard for purifying microvesicles and should quell any contamination concerns.

As mentioned previously, a number of tumor cells commonly shed ELMs into the peripheral circulation [55], [56]. It has also been demonstrated that ELMs exist within salivary secretions and carry transcriptomic information [42]. Although the origin of salivary ELMs remains unknown, here we provide evidence for their etiology by illustrating that peripherally secreted ELMs traverse the vasculature and land on oral cavity. We reveal this phenomenon by verifying that existence of seven salivary mRNA markers [57] in the malignant pleural effusion derived ELMs under same clinical setting (Table S2 in Supporting information S1). Four of these markers (BRAF [58], EGFR [59], LZTS1 [60], and FGF19 [61], [62]) have been directly correlated with lung cancer development and progression. These findings implicate ELMs as carriers of tissue-specific biomolecules throughout the vasculature and within bodily fluids.

To effectively investigate our hypothesis, we found it most convincing to pursue an animal model whose ELM constituents would be undeniably discriminatory. Using xenograft mice for this purpose is an ideal way to establish the fundamentals of our proposed mechanism, as implanted H460A cells should release species-specific ELMs that contain human molecular information. Identifying these markers in murine blood and saliva provides sufficient credence for the concept of ELM-based biomarker discovery using saliva.

Despite the strength of our design, a number of issues must be considered when determining the collective significance of the experimental outcomes. First, although we revealed species-specific ELM protein and transcript in the blood and saliva of our xenograft mouse model, we cannot justifiably conclude that they are identical. In other words, the structure and molecular constituency of ELMs respectively derived from blood or saliva may be fundamentally unique. Blood-based ELMs and their contents may be altered prior to their subsequent introduction into the oral cavity. Although our observations are in agreement with previous reports [47], we concede that the data presented here does not eliminate or definitively describe the interplay of ELMs within salivary gland tissues. Comprehensive proteomic and transcriptomic characterizations of hCD63-GFP–positive ELMs found in bodily fluids are needed to shed light on these issues. Future studies using patient-derived lung cancer xenograft mouse model containing a variety of genetic aberrations, and comprehensively proteomic and transcriptomic characterizations of blood and saliva ELMs could further our understanding of the role of ELMs in the circulation of tumor-specific bimolecules as well as the biogenesis of salivary biomarkers.

Second, detecting ELMs in oral fluids presented a substantial obstacle. Current assays, including Western blotting and acetylcholinesterase assay, proved unreliable owing to the small volumes of blood and saliva samples that are obtainable from murine subjects. Our solution, EFIRM, involves two antibodies that bind to non-overlapping hCD63-GFP fusion protein [45]. Because ELMs are commonly characterized by their CD63 membrane-bound proteins, there is an inherent concern of non-specific binding by murine ELMs to anti-human CD63 magnetic beads. To address this issue, we tested the specificity of EFIRM and found that mouse salivary ELMs produced a signal similar to background [45]. This outcome substantiates the specificity of this technique, and more importantly supports our aforementioned EFIRM data indicating the existence of human-specific molecules in the saliva and blood of our xenograft mouse model.

When the data is considered in its totality, one interesting value presents itself as somewhat of an aberration. Two tumor-bearing mice had human GAPDH mRNA in their saliva, but not their blood. While we speculate that distant pathologies secrete ELMs into the vasculature, these data insinuate that might not be the case. One possible explanation for this inconsistency may be the location of the tumorigenic cells. As it turns out, sputum (a respiratory expectorate used in lung cancer biomarker analysis [63], [64]) might be a means of bypassing the circulatory system. In this scenario, cancer cells or microvesicles could travel to the oral cavity via the pharynx, leaving the blood absent of disease-specific markers. This eventuality may be enhanced by the anatomical position of the neoplasm within the lungs. Hence, a lack of blood-derived lung cancer markers would not disqualify our model; on the contrary, it would strengthen the suggested efficacy of oral fluids as a diagnostic medium. In any case, additional investigations will be required to delineate the communicative interaction between the oral cavity and distant diseases.

Although our report does not definitively explain the etiology of salivary biomarkers, here we describe the detection of tumor cell-specific molecules in ELMs derived from saliva. Overall, our data suggest that ELMs released from distant tissues are involved in this process by serving as protective conduits of biochemical information. Considering this information, we put forth the idea that saliva is an effective source of discriminatory biomarkers and suggest that salivary ELMs are instrumental in their presentation.

## Supporting Information

Supporting information S1 **Supporting information, figure and tables.**
(DOC)
