# Supplementary material for: Detection of Tumor Cell-Specific mRNA and Protein in Exosome-Like Microvesicles from Blood and Saliva
Source: PLoS One. 2014 Nov 14;9(11):e110641. doi: 10.1371/journal.pone.0110641 (PMC4232306; doi:10.1371/journal.pone.0110641)
Supplement: Supporting information S1 — Supporting information, figure and tables. (DOC) [file pone.0110641.s001.doc]

**Supporting Materials and Methods**

#### Ethics Statement

Human pleural effusion samples were obtained from recently diagnosed and untreated lung cancer patients under the institutional review board protocol (IRB#10-000505) approved by the University of California Los Angeles IRB. Written informed consent forms were obtained from all participants.

Pleural Effusion ELMs Isolation

ELMs were isolated from pleural effusion using Exoquick precipitation solution (System Biosciences, Inc.). 300ųl of cell-free saliva was thoroughly mixed with 70ųl Exoquick solution, incubated at 4°C overnight, and centrifuged at 1500 × *g* for 15 min at 4°C. The pellets were then resuspended in water and used for RNA isolation.

Transcriptomic Profiling and Data Analysis

Unstimulated saliva samples were obtained from three healthy volunteers and processed according to previously established protocols . Saliva RNA was isolated from 330 µl of cell-free saliva supernatant and salivary ELMs using an RNeasy Mini Kit (Qiagen, USA) with the manufacturer’s instructions. Extracted RNA was linearly amplified using a RiboAmp RNA Amplification kit (Molecular Devices, Sunnyvale, CA). After purification, cDNA was transcribed and biotinylated using GeneChip Expression 3′-Amplification Reagents for *in vitro* transcription labeling (Affymetrix, Santa Clara, CA). Chip hybridization and scanning was performed at the UCLA microarray core facility using the Affymetrix Human Genome U133 Plus 2.0 array. All Affymetrix Human Genome U133 Plus 2.0 Array data generated in this study have been uploaded to the GEO database (http:www.ncbi.nlm.nih.gov/geo). The Accession number is GSE50700.

A probe set was defined as present when it had a P-value < 0.005 and an intensity value > 200. Ontological analysis was performed using the gene ontology analysis tool (http://www.geneontology.org/GO.tools.microarray.shtml).

RT-qPCR Assay

A 2-step RT-qPCR [reverse transcription followed by a separate qPCR step] was performed to detect salivary and ELMs-derived mRNAs. Multiplex RT-PCR preamplification of 8 mRNAs was performed using a SuperScript III platinum qRT-PCR System (Invitrogen) and a GeneAmp PCR-System 9700 apparatus (Applied Biosystems). Gene targets were quantitated using 2× SYBR Green qPCR Mastermix (Applied Biological Materials) with inner primers (900 nmol/L) and 2 μL cDNA template. Each gene was tested in triplicate for all samples. The primer sequences used are listed in Table S1.

**Table S1**

**Table S2:** qRT-PCR of US lung cancer salivary mRNA biomarkers

in pleural effusion derived ELMs

| **Gene** | **PE1** | **PE2** | **PE3** |
| --- | --- | --- | --- |
| FSR2 | D | ND | D |
| BRAF | D | D | D |
| LZTS1 | D | ND | D |
| FGF19 | D | D | D |
| EGFR | D | D | D |
| CCN1 | D | D | D |
| GREB1 | D | D | D |

**
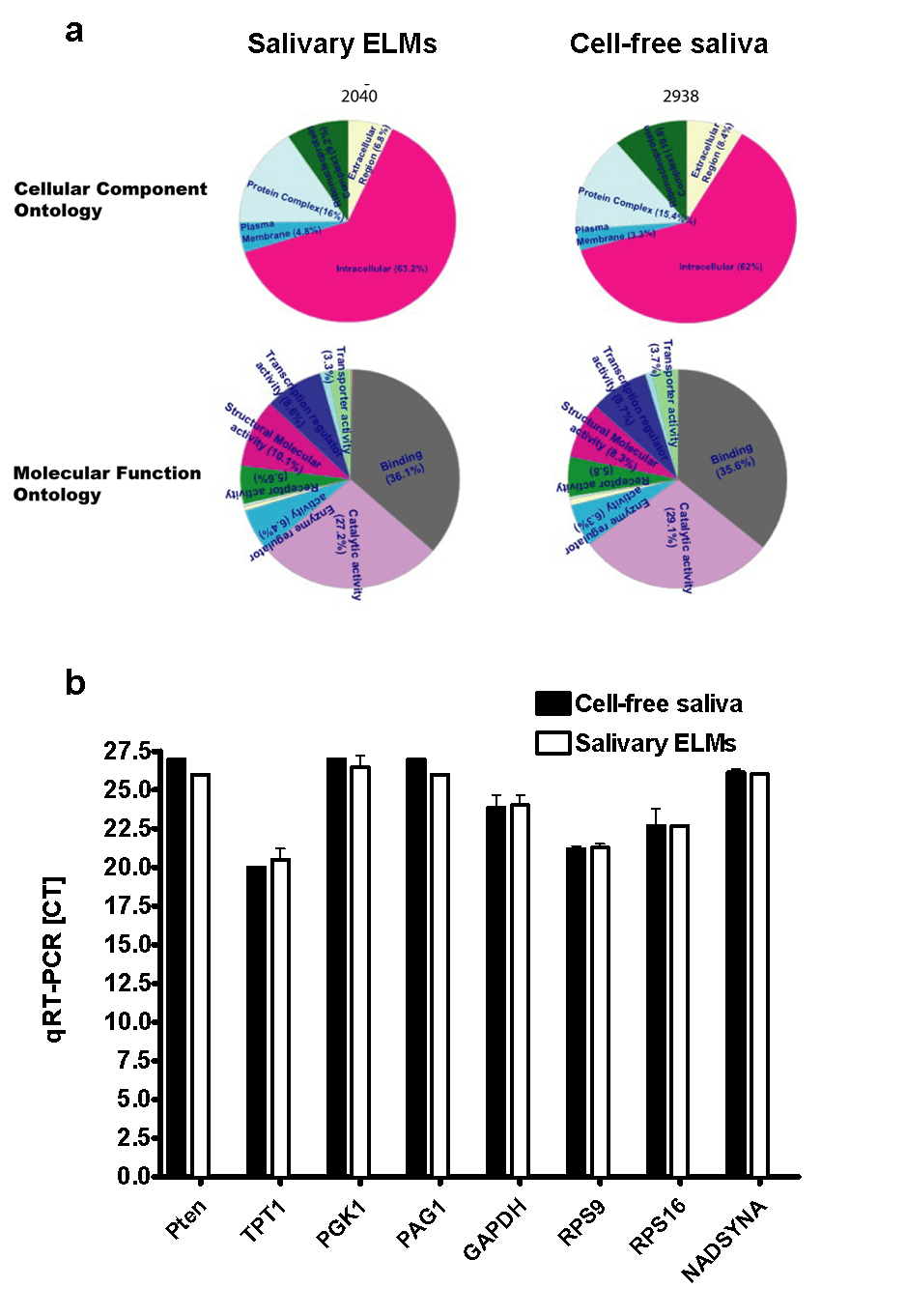
**

**Figure S1** Side-by-side comparison of the mRNA composition of salivary ELMs and cell-free saliva. (a) RNA from 300  μl of cell-free saliva and matched salivary ELMs were processed for Affymetrix HU133 Plus 2.0 microarray analysis (n=3). Shared mRNA species in all three samples were further classfied by cellular component and molecular function on the basis of their ontological descriptions. (b) RNA from 300 μl of cell-free saliva and matched salivary ELMs were processed for RT-qPCR analysis (n=7) using mRNA targets selected based on their abundance (microarray data).

**References**

1. Li Y, St John MA, Zhou X, Kim Y, Sinha U, et al. (2004) Salivary transcriptome diagnostics for oral cancer detection. Clin Cancer Res 10: 8442-8450.
